# Improvement of Targeted Chemotherapy of HER2-positive Ovarian Malignant Cell Line by Z_HER2_-Idarubicin Conjugate: An in vitro Study

**DOI:** 10.30699/IJP.2020.120392.2310

**Published:** 2020-12-20

**Authors:** Leila Siavoshinia, Mostafa Jamalan, Majid Zeinali, Aminollah Pourshohod, Mahdie Koushki, Bahman Moradipoodeh, Ghorban Mohammadzadeh

**Affiliations:** 1 *Department of Clinical Biochemistry, Faculty of Medicine, Ahvaz Jundishapur University of Medical Sciences, Ahvaz, Iran*; 2 *Department of Biochemistry, Abadan Faculty of Medical Sciences, Abadan, Iran*; 3 *Biotechnology Research Center, Research Institute of Petroleum Industry (RIPI), Tehran, Iran*; 4 *Hyperlipidemia Research Center, Department of Clinical Biochemistry, Faculty of Medicine, Ahvaz Jundishapur University of Medical Sciences, Ahvaz, Iran *

**Keywords:** Idarubicin, ZHER2 affibody, Idarubicin-ZHER2 affibody conjugate, Ovarian Cancer

## Abstract

**Background & Objective:**

Overexpression of human epidermal growth factor receptor 2 (HER2) causes cell transformation and development of various types of malignancies. Idarubicin is an effective anti-neoplastic drug but its specific delivery to the targeted cells is still a great challenge. Affibody as a cost-effective peptide molecule with low molecular weight has a high affinity for HER2 receptors. Breast and ovarian cancers as wide speared types of malignancies are associated with high expression of HER2. In the current study, we assessed the cytotoxic effects of idarubicin-Z_HER2_ affibody conjugate on the positive-HER2 cancer cell lines.

**Methods:**

The cytotoxic effects of constructed idarubicin-Z_HER2_ affibody conjugate on the SK-BR-3, SK-OV-3, and MCF-7 cells with various levels of HER2 expression were evaluated by MTT assay following 48 hours of incubation.

**Results:**

Idarubicin showed a potent and dose-dependent cytotoxic effect against all treated cell lines while the SK-OV-3 cells were significantly more sensitive. The dimeric form of the Z_HER2_ affibody molecule showed a mild effect on the cell viability of all treated cells at its optimum concentration. The constructed Idarubicin-Z_HER2_ affibody conjugate decreased the viability of SK-OV-3 cells at its optimal concentration, more efficiently and specifically than other treated cells.

**Conclusion:**

The Z_HER2_-affibody conjugate of idarubicin has a more specific cytotoxic effect compared with idarubicin alone against HER2-overexpressing ovarian cancerous cells. It appears the Z_HER2_-affibody conjugate of idarubicin has great potential to be implicated as an innovative anti-cancer agent in future clinical trials in patients with HER2-overexpressing ovarian cancer.

## Introduction

Ovarian cancer is one of the three major gynecologic malignancies and the most lethal among women worldwide (1). Surgery and chemotherapy based on platinum-based agents are the most commonly available protocols for the treatment of ovarian malignancies but the occurrence of drug-resistance and relapse of the malignancy in many cases could result in short-term survival of affected patients (2). Recently, there are several new drugs that have been tested in the clinical trials to evaluate their efficacy for the treatment of ovarian cancers (2). Furthermore, evaluation of the effect of various kinds of targeted therapy for the cure of epithelial ovarian cancer (EOC) is under active investigation (3). Among several markers that have been defined for EOC, DNA damage and repair system factors, molecules involved in the G1 cell cycle, PI3K pathway, MAPK pathway, and also growth factors attracted more attention for targeted therapy (3). Higher expression of epidermal growth factor receptors (EGFRs) in human ovarian cancers was found to be associated with shorter disease-free and overall survival. None of the designed EGFR inhibitors to date have shown promising results in clinical trials for successful treatment of patients with ovarian cancer. Unsatisfactory results were also obtained with the recombinant humanized monoclonal antibody, pertuzumab (Perjeta), that target specifically the extracellular domain of HER2 (4). The average 5-year relative survival rate for all types of ovarian cancer is about 47% (5). Thus, in the absence of a satisfactory ovary cancer cure protocol, the development of new drugs to improve the efficacy of cancer treatment and to increase the patient’s survival rate seems to be necessary. 

One of the most substantial targets in human malignancy targeted therapy is EGFRs (6). This tyrosine kinase family group includes four closely related receptors: HER1/ErbB1, HER2/ ErbB2, HER3/ErbB3, and HER4/ErbB4, which have several important roles in the growth and development of human normal cells (6). The HER2 receptor is highly expressed in various types of malignancies and its overexpression is associated with poor prognosis, cell transformation, tumor progression, angiogenesis, and metastasis of malignant cells (7). Therefore, HER2 has been used as a target for specific therapy in several solid tumors including breast, gastric, ovarian, lung, prostate, and bladder cancers (8-12). Overexpression frequency of HER2 in invasive epithelial ovarian cancer was reported 1.8% to 76% (13). There are some reports about HER2-targeted therapy of human ovarian cancer and several possible benefits of HER2-targeted immunotherapy have been discussed (13). Conjugation of monoclonal antibodies with a cytotoxic drug (antibody-drug conjugates or ADCs) by reducing the dose of chemotherapeutic agents is a promising approach in the treatment of patients with cancer (14, 15). These chimeric structures can deliver conjugated drugs to the targeted cells more selectively and efficiently (16). Alternatively, the antibody moiety of ADCs could be replaced with a low molecular weight affibody molecule. Affibodies are a new class of engineered affinity proteins that originated from the B-domain of the immunoglobulin binding region of staphylococcal protein A (17). Due to several advantages including low molecular weight, robustness, and thermostability, affibody is a valuable peptide molecule for targeted drug therapy and also tumor imaging (18-20). Affibody Z_HER2_ has a great affinity for the HER2 receptor and previously has been used for the delivery of various anti-cancer drugs to HER2-overexpressed cancerous cell lines (20-22). Our previous in vitro study indicated that idarubicin-Z_HER2_ affibody conjugate could be successfully used for specific ablation of HER2-overexpressed HN-5 cells originated from head and neck squamous cell carcinoma (HNSCC) (23). In the current study, the cytotoxic effects of idarubicin and idarubicin-Z_HER2_ affibody conjugate on two different HER2-overexpressed cell lines, ovarian malignant cells (SK-OV-3) and breast cancer cells (SK-BR-3), were compared to MCF-7 as a low expressing HER2 cell line. 

## Material and Methods

**Chemicals**

Idarubicin was purchased from Selleckchem (Houston, United States). Sulfosuccinimidyl 4-(N-maleimidomethyl) cyclohexane-1-carboxylate (Sulfo-SMCC), HisPur™ Ni-NTA resin, imidazole, and β-D-1-thiogalactopyranoside (IPTG) were obtained from Thermo Fisher Scientific (Massachusetts, United States). Ethylenediaminetetraacetic acid (EDTA), ampicillin, phenylmethanesulfonyl fluoride (PMSF), 3**-**[4, 5, dimethylthiazol-2-yl]-2, 5-diphenyl tetrazolium bromide (MTT), and Dulbecco's modified Eagle's medium (DMEM) were purchased from Sigma-Aldrich (St. Louis, MO, USA). The human HER2-positive cell lines including ovarian carcinoma cell line (SK-OV-3), and breast cancer cell lines (SK-BR-3, and MCF-7) were purchased from the National Cell Bank of Iran (Tehran, Iran). All other chemicals were obtained from Merck (Darmstadt, Germany).

**Expression and Purification of Z**_her2_ **Affibody **

Affibody Z_HER2 _gene was synthesized by *de novo* gene synthesis and one cysteine codon was also added to the 5΄ end of the gene (24). The recombinant Z_HER2 _affibody gene was inserted into a Champion™ pET302/NT-His plasmid from Invitrogen™ (Thermo Fisher Scientific) and cloned in competent *E. coli *BL21 cells. Z_HER2_ Affibody was expressed, purified, and qualified as previously described elsewhere (23). Briefly, the cultured cells that contained recombinant plasmid were centrifuged and the bacterial pellet was sonicated in lysis buffer (25 mM Tris-HCl, 1.0 mM EDTA and 1.0 mM PMSF, pH 6.8) by using a probe sonicator (UP50H, Hielscher, Teltow, Germany). After centrifugation of the cell lysate, the supernatant was collected for affibody purification. Purification of recombinant Z_HER2_ affibody was performed on the HisPur™ Ni-NTA resin (Thermo Fisher Scientific) according to the manufacturer's protocol. Finally, protein fractions were examined using coomassie brilliant blue (CBB) staining following SDS-PAGE to confirm the purity of the prepared affibody. Finally, affibody-containing fractions were pooled and protein concentration was determined using Bradford reagent (25). 

**Construction of Idarubicin-Z**_her2_ **Affibody Conjugate**

The idarubicin-Z_HER2_ affibody conjugate was constructed using sulfosuccinimidyl 4-(N-maleimidomethyl) cyclohexane-1-carboxylate (Sulfo-SMCC) as a heterobifunctional crosslinker as previously described (23). The Sulfo-SMCC allows covalent conjugation of amine- and sulfhydryl-containing molecules through the formation of stable thioether bonds. Briefly, DMSO-dissolved Sulfo-SMCC diluted in conjugation buffer (PBS, 2.0 mM EDTA, pH 7.2) and added to the idarubicin solution (1.0 mg mL^-1^) at a 2-fold molar excess. The reaction mixture was incubated for 30 min at room temperature and then affibody solution was added to the mixture in a 1:1 molar ratio. After 30 min, unreacted cross-linker and unconjugated idarubicin molecules were separated from Idarubicin-Z_HER2_ affibody conjugate using a desalting column equilibrated with conjugation buffer. The product was concentrated using Amicon Ultra-15 centrifugal filter unit. Construction of idarubicin-Z_HER2_ affibody conjugate was confirmed using a UV-Vis spectrophotometer (SPECORD 210 plus, Analytik Jena, Germany) and fluorescence spectrophotometer (LS-45 Fluorescence Spectrometer, Perkin-Elmer, USA) as previously described (23). 

**Cell Viability Assay**

The SK-BR-3 and MCF-7 cell lines were cultured in complete DMEM with high glucose, and the SK-OV-3 cell line was cultured in complete RPMI-1640 (with high glucose) under a humid atmosphere (37°C, 5% CO_2_). The cell viability was measured by MTT colorimetric assay. Briefly, the cells in the logarithmic growth phase were seeded at approximately 1×10^4 ^cells well^−1^ into flat-bottom 96-well plates (Nunc, Roskilde, Denmark). The cells were treated with various concentrations of idarubicin (0.001-0.1 µM), Z_HER2_ affibody (0.1-100 µM), and idarubicin-Z_HER2_ affibody conjugate (0.01-50 µM) for 48 hours in triplicate. Then, the culture medium was replaced with fresh medium and MTT solution was added and incubation continued for another 4.0 hours. After washing the cells three times with PBS the pure DMSO (200 μL) was added. The absorbance of each well was read at 570 nm using a microplate reader (BioTek ELX800, USA). 

**Determination of Her2 Expression Level in Cancerous Cell Lines**

Western blot analysis was performed for comparison of HER2 expression in three investigated cancerous cell lines. The cells at a density of 5×10^5^ were plated into the 10-cm dishes, incubated at 37°C overnight, and then homogenized in RIPA buffer (10 mM Tris-HCl, pH 8.0, 0.1% Triton X-100, 3% SDS, 1.0 mM EDTA and 150 mM NaCl) supplemented with protease inhibitors. The cell lysate was centrifuged at 10,000 g for 15 min at 4°C and protein concentration of the supernatant was determined using a bicinchoninic acid (BCA) protein quantitation kit. The samples (50 μg each) were electrophoresed on 12% SDS-PAGE, and then were electro-transferred onto polyvinylidene difluoride (PVDF) membranes (Millipore, US). After blocking with 5% skimmed milk-containing TBS-T solution (Tris-buffered saline with 0.1% Tween 20) for 2 hr at room temperature, primary anti-HER2 antibody (Abcam, Cambridge, UK) was added and incubation was processed overnight at 4°C. After washing the blotted membrane three times with TBS-T and incubation with HRP-conjugated secondary antibody for 1 hr, protein bands were visualized with a Chemi-Doc gel documentation system (Bio-Rad, Hercules, CA, USA) by enhanced chemiluminescence (ECL) kit (Bio-Rad, Hercules, CA, USA). The intensity of bands was quantified by densitometry analysis of blots using Image J software and described as a ratio to β-actin expression.

**Statistical Analysis**

All experiments were performed in triplicate. All data were processed using SPSS software version 15 (SPSS Inc., Chicago, IL., USA) and expressed as mean ± SEM. Data were analyzed by ANOVA to determine differences between experimental groups. A P-value <0.05 was considered as statistically significant. 

## Results

**Affibody Z**_HER2_ **Expression**

**Fig. 1:**
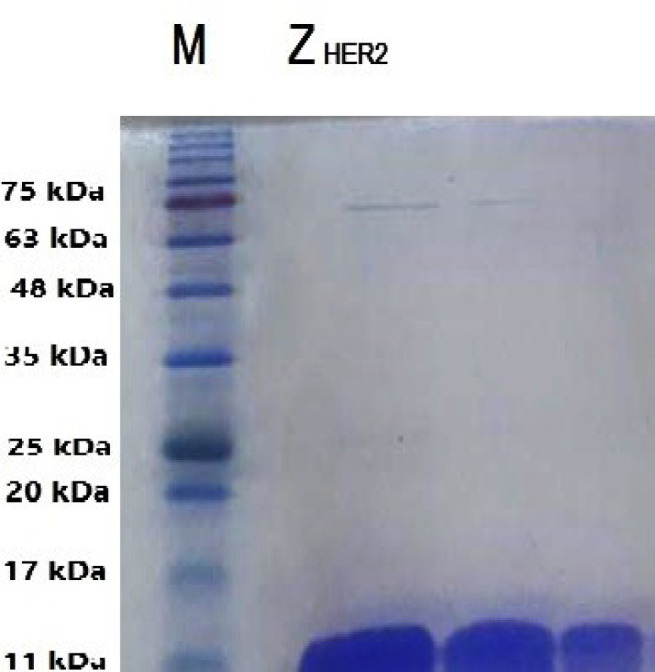
Purified Z_HER2_ affibody molecule was analyzed by SDS-PAGE

In the current study, BL21 (DE3) competent *E. coli* as a bacterial host was used for cloning and expression of the engineered Z_HER2 _affibody gene. Affibody molecules were purified using Ni-affinity column chromatography and the purity of the product was assessed by the SDS-PAGE analysis. As shown in Figure 1, a single protein band with a molecular weight of about 14 kDa was observed after staining of acrylamide gel with CBB.

The protein bands were stained with Coomassie brilliant blue. A single sharp band with a molecular weight around 14 kDa confirmed the purity of the affibody product. 

**HER2 Expression in Different Cancerous Cell Lines**

**Fig. 2:**
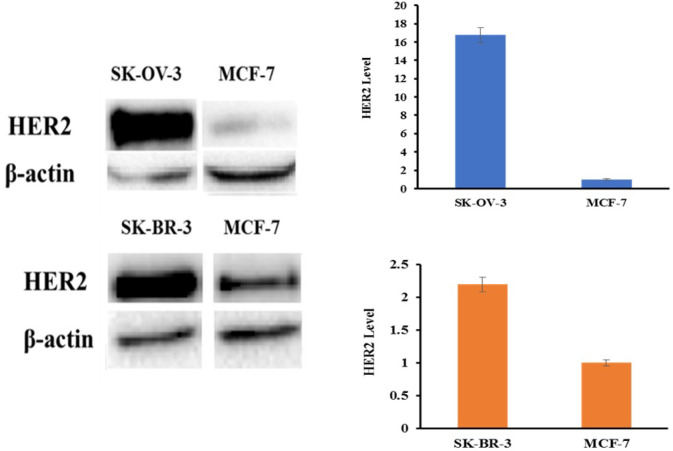
Western blots analysis of HER2 expression in different cancerous cell lines

The level of HER2 expression in three different cancerous cell lines was evaluated using western blot analysis. Our results indicated that the level of HER2 expression in SK-OV-3 cells was significantly higher than the other examined cell lines as shown in Figure 2. 

Over-expression of HER2 in SK-OV-3 and SK-BR-3 cells compared with MCF-7 cell line was verified using immunoblotting. As shown, SK-OV-3 and SK-BR-3 cell lines express HER2 at a higher level compared with MCF-7 cells with normal expression of HER2.

**Cytotoxic Effect of Idarubicin on the Cancerous Cell Lines**

**Fig. 3:**
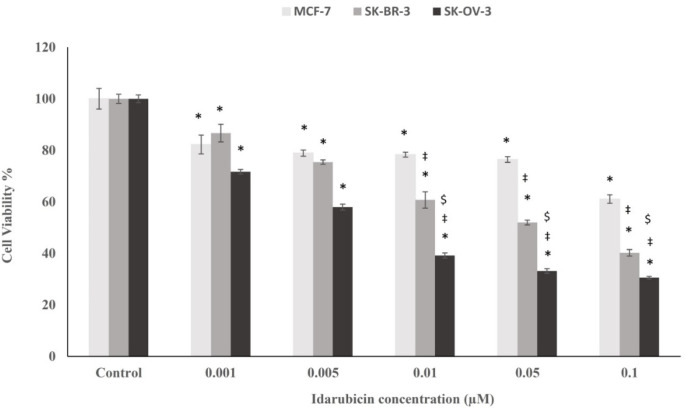
Effect of various concentrations (0.001-0.1 µM) of idarubicin on the cell viability of MCF-7, SK-BR-3, and SK-OV-3 cells

To our knowledge, there is no report about the cytotoxic effects of idarubicin either alone or with its conjugate with affibody on the SK-OV-3 cancerous cell line. In the current study, HER2-positive SK-OV-3 cells were treated with idarubicin at a concentration range between 0.001 to 0.1 µM and the cell viability was compared to SK-BR-3 and MCF-7 cell lines that were treated in a similar way (Figure 3). Based on the obtained results, idarubicin could considerably decrease the cell viability of SK-OV-3, MCF-7, and SK-BR-3 cells in a dose-dependent manner after 48 hours of incubation in the indicated conditions (Figure 3). At the highest examined concentration of idarubicin (0.1 µM), the viability of MCF-7, SK-BR-3, and SK-OV-3 cells were 61.09%, 40.21%, and 30.55%, respectively. Our results demonstrated that the SK-OV-3 cell line is more sensitive to idarubicin in comparison to SK-BR-3 and MCF-7 cells. .

*P-value<0.05 in comparison to the corresponding control group, ‡ P-value<0.05 in comparison to the MCF-7 cells treated with idarubicin at the same concentration, $ P-value<0.05 in comparison to the SK-BR-3 cells treated with idarubicin at the same concentration.

**Cytotoxic Effect of Z**_her2_ **Affibody and Idarubicin-Z**_her2_ **Affibody Conjugate on the Treated Cancerous Cells**

**Fig. 4:**
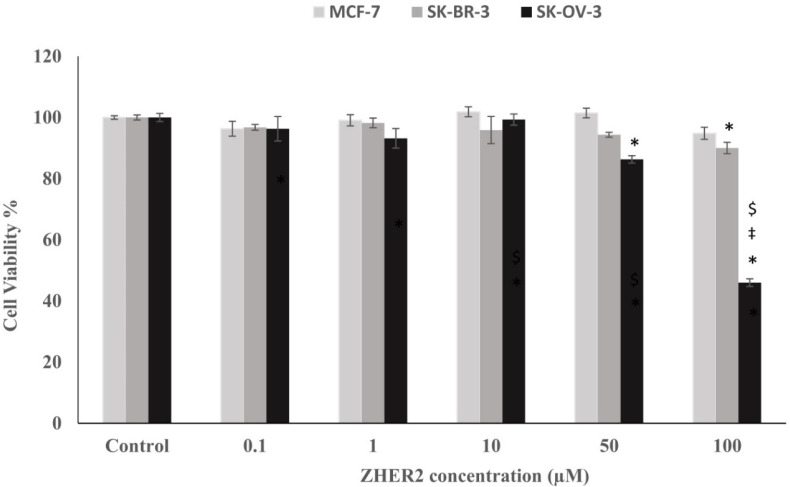
Effect of various concentrations (0.1-100 µM) of purified Z_HER2_ affibody on the cell viability of MCF-7, SK-BR-3, and SK-OV-3 cells

In the current study, three different cancerous cell lines, SK-OV-3, SK-BR-3, and MCF-7 cells were treated with different concentrations (0.1-100 µM) of purified Z_HER2_ affibody (Figure 4). After 48 hours of treatment with Z_HER2 _affibody the cell viability of SK-OV-3, SK-BR-3, and MCF-7 cells at the highest examined concentration (100 µM) was 45.98%, 90.01, and 94.81 compared to untreated control cells (Figure 4). The elevated level of HER2 expression on the surface of SK-OV-3 cells in comparison to SK-BR-3 and MCF-7 cells may explain the more sensitivity of this cell line to Z_HER2_ affibody. 

**Fig. 5:**
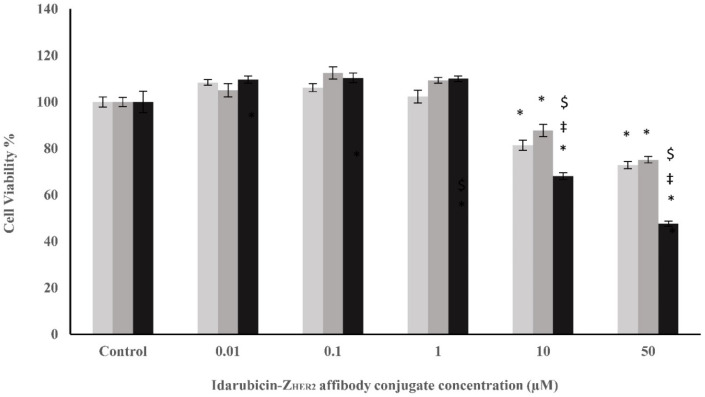
Effect of constructed idarubicin-Z_HER2_ affibody conjugate on the cell viability of SK-OV-3, SK-BR-3, and MCF-7 cells

In the current study, for the construction of an immuno-conjugate drug, Z_HER2_ affibody was covalently bound to idarubicin through a heterobifunctional sulfo-SMCC crosslinker. The Sulfo-SMCC linker permits covalent conjugation of amine-containing (idarubicin) and sulfhydryl-containing (Z_HER2_ affibody) molecules. The formation of the immuno-conjugate was confirmed by UV-Vis and fluorescence spectrophotometry as previously described (23). The effect of idarubicin-Z_HER2_ affibody conjugate on the cell viability of SK-OV-3, SK-BR-3, and MCF-7 cell lines was also investigated (Figure 5). Our results indicated the constructed conjugate at both concentrations of 10 and 50 µM could significantly reduce the cell viability of all three examined cell lines. After 48 hr of incubation time in the presence of the conjugate at a concentration of 50 μM, the cell viability of MCF-7, SK-BR-3, and SK-OV-3 cells was reduced to 72.89, 75.16, and 47.61%, respectively. As seen in Figure 5, the conjugate is more efficient in reducing the viability of SK-OV-3 cells compared with MCF-7 and SK-BR-3 cells. 

* P-value<0.05 in comparison to the corresponding control group, ‡ P-value <0.05 in comparison to the MCF-7 cells treated with Z_HER2_ affibody at the same concentration, $ P-value<0.05 in comparison to the SK-BR-3 cells treated with Z_HER2_ affibody at the same concentration.

* P-value<0.05 in comparison to the corresponding control group, ‡ P-value<0.05 in comparison to the MCF-7 cells treated with idarubicin-Z_HER2_ affibody conjugate at the same concentration,

$ P-value<0.05 in comparison to the SK-BR-3 cells treated with idarubicin-Z_HER2_ affibody conjugate at the same concentration.

## Discussion

Affibody, a 58-amino acid protein domain was originally derived from one of the IgG-binding domains of Staphylococcal Protein A (SpA) which is one of the important and fascinating molecules in life sciences for therapeutic, diagnostic imaging, and biotechnological applications (24). Recently, clinical trials of specific affibodies and its efficacy for determining HER2 status in metastatic breast cancer have been investigated by Sorensen* et al.* [26]. They found that the useful and cost-effectiveness affibody complex without meaningful adverse effect was well tolerable for the detection of several metastatic lesions with HER2 over-expression. Although the anti-proliferative effect of idarubicin either alone or in combination with other chemotherapeutic agents has been studied on the MCF-7 and HN-5 cell lines and its cytotoxicity mechanism has been completely determined previously (26 -28), to the best of our knowledge, there is no report about the cytotoxic effects of idarubicin either alone or in combination with affibody on the SK-BR-3 and SK-OV-3 cell lines. Therefore, we investigated the effect of a covalent construct of idarubicin-Z_HER2_ affibody conjugate on the SK-OV-3, SK-BR-3, and MCF-7 cell lines for specific targeting therapy of malignant cells with overexpressing of HER2 compared with a malignant cell line with normal expression of HER2 as a control group.

At the first step, Z_HER2_ affibody was expressed and purified on SDS-PAGE as shown in Figure 1. The purified Z_HER2_ affibody showed a molecular weight of approximately 14 kDa which is consistent with the previously published results (18, 20, 23, 24). In the conjugated molecule, affibody was covalently bound to idarubicin through a heterobifunctional sulfo-SMCC crosslinker. The Sulfo-SMCC linker permits covalent conjugation of amine-containing (idarubicin) and sulfhydryl-containing (Z_HER2_ affibody) molecules which can be confirmed by using UV-Vis and fluorescence spectroscopy as previously described elsewhere (20). Then, the expression of HER2 was determined in three cancerous cell lines. Although, the expression of HER2 in different cancerous cells can be assessed by different methods including western blotting (WB), immunohistochemistry (IHC), fluorescence in situ hybridization (FISH), chromogenic in situ hybridization (CISH), and enzyme-linked immunosorbent assay (ELISA) (11), in the current study, the expressions of HER2 in three different cancerous cell lines were evaluated by western blotting. Our results indicated the expression of HER2 in SK-OV-3 cells was higher than the other cell lines consistent with the previously published results reviewed by Iqbal* et al.* (29).

Finally, idarubicin either alone or in combination with affibody had a significant cytotoxic effect on the cell viability of SK-BR-3, SK-OV-3, and MCF-7 cancerous cell lines. The anti-proliferative effect of idarubicin either alone or in combination with other chemotherapeutic agents has been studied in various types of cancerous cell lines such as MCF-7 and HN-5 and the mechanism of its cytotoxicity have been elucidated (30-32). Idarubicin (4-demethoxydaunorubicin) is a synthetic structural analog of anthracyclines that is extensively used for the treatment of human leukemia (33). Conversely, to other available anthracyclines, idarubicin has a significant oral bioavailability (34). Idarubicin binds non-covalently to DNA with a high affinity and inhibits DNA synthesis via interaction with topoisomerase II (35). Furthermore, anthracycline-iron complexes which are emerged in cells can induce oxidative stress that finally result in structural DNA damage, cellular membrane destruction, and p53-related apoptosis (36). Moreover, it is demonstrated that other pathways like mTOR-related cytotoxic autophagy can be induced by idarubicin in leukemia cancerous cells (37). 

To our knowledge, there is no report about the cytotoxic effects of idarubicin either alone or as conjugated to affibody on SK-OV-3 cancerous cell line. In the current study, HER2-positive SK-OV-3 cells were treated with idarubicin at a concentration range between 0.001 to 0.1 µM and cell viability was compared to SK-BR-3 and MCF-7 cell lines treated with the similar concentrations (Figure 3). Based on the obtained results, idarubicin could significantly decrease the cell viability of SK-OV-3, MCF-7, and SK-BR-3 cells in a dose-dependent manner after 48 hours of incubation at the indicated conditions (Figure 3). Today, many ovarian malignancies are still treated with platinum-based chemotherapeutic agents, which can affect all cancerous and healthy cells and tissues as non-specific (38). Platinum-based antineoplastic drugs induce their effect through DNA crosslinking and interfering with DNA repair mechanisms which consequently lead to DNA damage and apoptosis in all treated cell types (39). In contrast, idarubicin specifically targets topoisomerases that are active in proliferative cells (40), and therefore, its function in targeting malignant cells is more specific compared with common platinum therapy.

In the present study, three different cancerous cell lines including SK-OV-3, SK-BR-3, and MCF-7 were treated with different concentrations (0.1-100 µM) of purified Z_HER2_ affibody (Figure 4). Our results indicated that the purified Z_HER2_ affibody at the 50 and 100 µM could significantly decrease the cell viability of SK-OV-3 cells compared to SK-BR-3 and MCF-7 cells. Previously, Z_HER2_ affibody has been used for imaging of HER2-positive tumors (28, 41, 42). Ekerljung *et al.* reported Z_HER2_ affibody in dimeric form could reduce the cell viability of HER2-overexpressing SK-BR-3 cells (43). They found that the cytotoxic effect of dimeric Z_HER2_ affibody on the SK-BR-3 cell line is comparable to trastuzumab (Herceptin®) as an FDA-approved therapeutic monoclonal antibody routinely used for the treatment of patients with HER2-positive breast tumors (43). 

Immuno-conjugates commonly are constructed from a targeting moiety, whole antibody molecule or its Fab domain, and a toxic part which is considered as promising therapeutic agents that have been attracted more attention in recent years for cancer therapy. Immuno-conjugate drugs are under active investigation for specific induction of apoptosis in malignant cells and some of them have been approved by the FDA for the treatment of cancer patients (44). Due to some limitations in the application of antibodies or their Fab fragments, affibody molecules introduced with exceptional characteristics such as low molecular weight and high affinity were offered as new tools for designing new immunotoxins against cancerous cells (44, 45). After successful usage of anti-HER2 affibody molecules in high contrast imaging of HER2-positive human tumors, these new engineered molecules were applied for the specific delivery of anticancer agents (46, 47). Alavizadeh *et al.* confirmed that HER2 affibody-cisplatin containing liposomes (affisomes) could improve the therapeutic activity of cisplatin in a HER2-overexpressing tumor model (47). In the current study, for the construction of an immuno-conjugate drug, Z_HER2_ affibody was covalently bound to idarubicin through a heterobifunctional sulfo-SMCC crosslinker. The Sulfo-SMCC linker permits covalent conjugation of amine-containing (idarubicin) and sulfhydryl-containing (Z_HER2_ affibody) molecules. The formation of the immuno-conjugate was confirmed by UV-Vis and fluorescence spectrophotometry as previously described (23). Our results indicated that the effect of idarubicin-Z_HER2_ affibody conjugate on the SK-OV-3 was significantly higher than the SK-BR-3 and MCF-7 cells (Figure 5). To our knowledge, this result is the first report about the cytotoxic effect of idarubicin-Z_HER2_ affibody conjugate on the cell viability of SK-OV-3 ovarian cancerous cells. Based on our results, we could propose the idarubicin-Z_HER2_ affibody conjugate as an anti-cancerous therapeutic agent should be more investigating for clinical trials in malignant patients with high expression of HER2 receptors, especially in ovarian cancer.

## Conclusion

Chemotherapeutic agents that are commonly used in the treatment of various human malignancies have several undesirable side effects. Approaches for specific delivery of antineoplastic drugs to malignant cells are seriously considered by drug discovery programs of pharmaceutical companies. In HER2-targeted therapy, as a new approach for the treatment of several human malignancies, therapeutic agents are directed toward HER2-positive cells by linking them to a peptide with a high affinity for the HER2 receptor. Idarubicin individually reduced the cell viability of all three examined cell lines in a dose-dependent manner but its effect on the SK-OV-3 cells was more potent. So, it seems idarubicin may be considered as a new medication for the treatment of ovarian tumors. On the other hand, and in regard to the higher expression of HER2 in SK-OV-3 cells, the more cytotoxic effect of Z_HER2_ affibody on this cell line is not out of mind. Finally, it seems that by conjugation of idarubicin to Z_HER2_ affibody, the specificity of idarubicin for inhibition of SK-OV-3 proliferation can be improved by facilitating its delivery into targeted cells. More investigations are needed to verify the idarubicin-Z_HER2_ affibody conjugate as a potential candidate for the treatment of HER2-overexpression ovarian malignancies.
